# The role of self-care in perceptions of satisfaction with life, organisational job satisfaction, and self-efficacy in zoo and aquarium professionals

**DOI:** 10.3389/fvets.2025.1677195

**Published:** 2025-12-05

**Authors:** Sabrina Brando, Vinícius Donisete Lima Rodrigues Goulart, Hannah M. Buchanan-Smith, Sonia Rey Planellas, Line Caes

**Affiliations:** 1 INTERBEING AnimalConcepts, Alicante, Spain; 2 Psychology, Faculty of Natural Sciences, University of Stirling, Stirling, United Kingdom; 3 Transportation Research and Environmental Modelling lab (TREM) - Institute of Geosciences, Federal University of Minas Gerais, Belo Horizonte, Brazil; 4 IRTA (Institute of Agrifood Research and Technology), Animal Welfare Program, Girona, Spain

**Keywords:** satisfaction with life, organisational job satisfaction, self-efficacy, self-care, zoo, aquarium, calling, education

## Abstract

This research sheds light on how professionals with different roles in zoos and aquariums perceive individual and organisational job satisfaction, self-efficacy, and satisfaction with life, and which types of self-care contribute to these variables. This was achieved through a mixed methods approach of a survey (completed by 442 participants) and follow-up interviews (in 39 participants) with recruitment across 23 organisations. For the single question, *I am satisfied with my job (reflecting individual job satisfaction)*, 44% of participants responded *somewhat agree* and 27% responded *strongly* agree, indicating that most participants (71%, *n* = 442) were satisfied with their job, and this was particularly true for those who considered their job a calling. Differences across job roles were found, with the largest variability for those in Junior animal caregiver positions, and with CEOs reporting the highest median of individual job satisfaction scores. A very weak positive significant relationship exists between self-efficacy and levels of organisational job satisfaction, which may be explained by the fact that this relationship is complex and multifaceted. A weak positive significant relationship between self-efficacy and satisfaction with life and a moderate positive relationship between organisational job satisfaction and satisfaction with life were also identified. The Generalised Linear Model analyses revealed that only one factor, *Experience*, and contributed to all independent variables (i.e., total satisfaction with life, mean organisational job satisfaction, and total self-efficacy), suggesting that participants with more time in the field reported higher total satisfaction with life, organisational job satisfaction, and levels of total self-efficacy. Various types of self-care (physical, emotional, spiritual, and workplace) were also significant positive contributors to satisfaction with life, but only workplace self-care contributed positively to organisational job satisfaction, whereas only intellectual self-care contributed positively to self-efficacy. Thematic analyses of the interview data contextualised the findings, with specific examples. This study contributes important empirical data that provides guidance for developing holistic employee wellbeing programs, continued professional development, and more appropriately aligned education to improve overall wellbeing of zoo and aquarium personnel.

## 1 Introduction

There is growing interest in employee wellbeing in zoos and aquariums [henceforth ZOAQ(s)], given the wide-ranging negative implications that poor wellbeing can have for both individuals and organisations. For example, while employees in wild animal care and conservation report their professions as meaningful and a calling (1, 2), research also shows such workers raise concerns regarding poor health, burnout, stress, and job-related stressors (1, 3–6). These factors can lead to high turnover rates, increased mistakes, significant costs, reduced productivity, and reduced job satisfaction (1, 7–9). Therefore, understanding how to promote positive and reduce negative employee wellbeing in ZOAQs has become a major concern and mission for both research and practice.

This research addresses a wide range of interconnected factors of wellbeing, including satisfaction with life (henceforth life satisfaction), individual and organisational job satisfaction, self-efficacy, calling, and the types of self-care zoo professionals engaged in. Each of these is reviewed, starting with wellbeing. To date, although there is no unified and agreed definition for wellbeing (10), and there is considerable variation in how researchers define, understand, explain, and especially measure the construct as made evident in a scoping review by Bautista et al. (11). One of the most widely cited definitions is: “wellbeing can be understood as how people feel and how they function both on a personal and social level, and how they evaluate their lives as a whole” (12), p. 6. Good wellbeing reflects the degree to which individuals feel fulfilled across various life domains, such as family, social relationships, work, financial, and environmental (13, 14). High individual and organisational job satisfaction can contribute to positive occupational wellbeing (15). Employees may experience high individual job satisfaction but still struggle with occupational wellbeing due to factors such as long working hours, support, or a lack of work-life balance (16), which in turn may reduce life satisfaction (17). Finances affect where people can live, safety, commute time impacting time with family, rest, opportunities to save, and so on, and different dimensions of wellbeing will have different challenges, opportunities, and spillovers. Therefore, it is essential to holistically consider individual wellbeing, an integrated approach considering personal and work life.

Life satisfaction is linked to, but conceptually different from, wellbeing and has been described as the extent to which a person finds their life rich, meaningful, full, or of high quality (18). It has been linked to increased psychological wellbeing (PWB) and subjective wellbeing (SWB). SWB and PWB are typically treated as distinct concepts (19, 20). SWB focuses on the individual’s subjective evaluation of their life, including the cognitive component of life satisfaction as a whole (21) and the balance between positive and negative emotions (22–24). PWB encompasses a broader sense of flourishing, denoting good mental and physical health (18), including autonomy, personal growth and relationships, purpose in life, and environmental mastery (19). While SWB is often linked to happiness and life satisfaction, PWB delves deeper into the development of individual potential and resilience, including positive mental health outcomes. One way of increasing life satisfaction is to create high-quality jobs where staff have more opportunities, feel respected and heard. For example, Batool et al. (17) reported bidirectional causality between life and job satisfaction, with job satisfaction having a greater effect on life satisfaction than vice versa.

Job satisfaction is a “pleasurable or positive emotional state, resulting from the appraisal of one’s job experiences” (25), p. 316. It includes individual and organisational aspects that affect organisational success, employee retention, and overall workplace morale. Individual job satisfaction refers to overall contentment with work and, while not optimal considering the complex construct, can be assessed with a single validated item (26). Organisational job satisfaction covers aspects like organisational support and employee wellbeing, and can assessed across multiple items (27). Job satisfaction is influenced by a variety of internal and external factors, including organisational culture, work-life integration, recognition, compensation, and professional development (28). Montuori et al. (29) found that job satisfaction reflects overall quality of life, and research shows a correlation between job and life satisfaction (17, 30). Dreer’s (31) survey found positive emotions strongly predict job satisfaction and retention. Individuals with positive emotions can set meaningful goals, maintain positive expectations, and overcome adversities (32). All of these are important attributes, skills, and states for any person in any profession, and perhaps more so in professions perceived as a calling due to the high levels of emotional and mental engagement and investments (33).

A calling is defined as a meaningful passion people experience towards a domain (34) and “…seeking a sense of overall purpose and meaning and is used to help others or contribute to the common good…” (35), p. 426. Most people entering zoo professions are committed to animal care, conservation, and perceive their job as a calling (1, 2, 36). These professions offer limited economic benefits or advancement opportunities and can result in reduced empowerment and job satisfaction (1). Zoo professionals find broader meaning in their work and willingly sacrifice money, time, and wellbeing (2). Bunderson and Thompson (2) found that while work provided meaning, it demanded sacrifice and duty, making professionals vulnerable to exploitation and associating calling negatively with income. The authors concluded that meaningful work can become a double-edged sword.

The ability to achieve a desired outcome, successfully accomplish specific tasks, or overcome challenges is referred to as self-efficacy. Self-efficacy refers to a person’s belief in their capability to successfully perform a particular task (37). This core belief is “the foundation of human inspiration, motivation, performance accomplishments, and emotional well-being, … and operates through its impact on cognitive, motivational, affective, and decisional processes” (38). Self-efficacy has been found to be directly related to autonomy satisfaction and competence satisfaction (39). Studies have found that workplace self-efficacy is related to life satisfaction, for example, among elementary school teachers, nurses, physicians, and a broad range of other workers (40–42). Furthermore, self-efficacy mediates a robust relationship between servant leadership and employee wellbeing, highlighting the importance of supportive leadership traits such as humility, servanthood, and integrity (43).

Key factors contributing to self-efficacy include an internal locus of control, a strong learning orientation, and favourable work environment characteristics such as management support and growth opportunities (44). Individuals with an internal locus of control and a focus on learning tend to exhibit higher self-efficacy (44), which can enhance their ability to effectively engage in self-care practices, as suggested by Bandura’s (45) Social Cognitive Theory. Self-care is defined as the ability to care for oneself through awareness, self-control, and self-reliance to achieve, maintain, or promote optimal health and wellbeing (46). For instance, physical exercise, social support, and relaxation have been associated with better wellbeing (47), and self-care across different domains has been proposed for attending to the whole person (48). Beyond the impact on general wellbeing, self-care practices have a significant impact on job satisfaction, and professional self-care is reported to be the most important but least utilised form of self-care (49). Furthermore, given their belief in their ability to manage stress and maintain work-life balance, employees with high self-efficacy are more likely to engage in self-care practices (50).

The potential negative impacts induced from workplace challenges, strains, and inequalities on life satisfaction and job satisfaction call for a holistic approach to employee wellbeing and ways to support and promote good wellbeing at all levels of the organisation. Organisations and leadership should provide support and resources to achieve collective goals, which Sabrina Brando, the first author of this manuscript (SB) calls a We-Care approach (51, 52). This includes for example, supporting teams and individuals to achieve desired outcomes, such as excellence in animal care and wellbeing, managing workload, role-modelling healthy self-care practices, and supporting those in more vulnerable positions.

Despite this growing evidence, there is a general lack of research on the wellbeing of employees in ZOAQs. Specifically, insufficient attention has been directed towards understanding how zoo employees perceive their life satisfaction, individual and organisational job satisfaction, and self-efficacy. Additionally, insights into the types of self-care these professionals engage in and how they relate to overall wellbeing are absent.

This research aims to shed light on how zoo professionals in diverse roles, including management and upper management positions, perceive their overall life satisfaction, organisational job satisfaction, and self-efficacy and whether different types of self-care contribute to these variables. This is essential to support people in mission-driven fields. Including employees from different levels throughout an organisation is of key importance in providing a broader range of perspectives. Specifically, we tested the following three hypotheses.

1. Individuals with high levels of self-efficacy report higher levels of individual job satisfaction & organisational job satisfaction
2. Individuals with high levels of self-efficacy report higher levels of satisfaction with life
3. Individuals with higher levels of satisfaction with life report higher levels of individual job satisfaction & organisational job satisfaction

Furthermore, we examined the types of self-care that correlated with higher levels of self-efficacy, life satisfaction, and organisational job satisfaction. As we did not have any pre-conceptualisation, we did not formulate hypotheses about the types of self-care contributing to the three variables and treated this as an exploratory evaluation.

## 2 Materials and methods

This project employed a mixed methodology design, approved by the General University Ethics Panel (GUEP) at the University of Stirling, Scotland, with reference number GUEP 2023 711,811,265. Two distinct methodologies (quantitative survey and qualitative interviews) for data collection were used to gain insights into individual, team, leadership, and organisational aspects of employee wellbeing in ZOAQs. This study was designed for online data collection due to COVID conditions. The web-based survey was administered through University of Stirling’s licence for Qualtrics. Online interviews were conducted using the University of Stirling’s licence for Microsoft Teams. The survey opened on 11 July 2023 and closed on 28 September 2023. All interviews, with a subset of participants who indicated interest, were conducted after the survey was closed between 26 October 2023 and 30 January 2024.

Olmos-Vega et al. (53) frame reflexivity as a way to embrace and value a researcher’s subjectivity, and SB believes a reflexivity exercise should be part of both quantitative and qualitative studies. SB coded all the interviews, next to analysing all the other data from the survey. She has worked in the zoo and aquarium fields for over 37 years, was the first director of animal wellbeing of the World Association of Zoos and Aquariums, and is the founder and CEO of the consultancy organisation AnimalConcepts. SB is a psychologist with a specialisation in Animal Studies. Her critical realism epistemological and unique viewpoints, close collaboration, reflections, combined with discussions with all co-authors shaped this research project and analysis. The study design and questions, data collection, and mixed-methods analysis embrace decades of experience, science, and practices, with the aim of reducing excessive bias and unfounded interpretation. The research questions and hypotheses guided this research project, and experience informed the appropriate and meaningful data analysis approaches.

Quotes have been used to contextualise the findings in the results section, as advised by Braun and Clark (54) and highlighted by Byrne (55) when discussing appropriate reporting styles and traditional reporting conventions. Throughout the discussion section, including the demographics, further quotes from the interviews were embedded to support the triangulation of all findings across the quantitative and qualitative data and to reflect the lived experiences of these participants, who will also be referred to as interviewee(s). Unless specifically stated, all quotes were obtained from this study.

## 3 Participants

### 3.1 Recruitment

Participating organisations were recruited from a subset of ZOAQs. Specific criteria to qualify included: (1) the facility must be accredited by a national or regional zoo and aquarium association; (2) the respondents are expected to read, write, and understand English well; and (3) the facility is midsize to large in staff numbers (at least 50 people). A total of 52 ZOAQs were approached with a letter of invitation which included a complimentary webinar to be delivered after participation. Of the 52 organisations that approached, 16 declined, 13 did not respond, and 23 accepted the invitation. There were seven participating countries: the United Kingdom (1), the United States of America (7), Canada (3), Australia (8), New Zealand (2), Singapore (1), and China (1). Accreditation associations included the European Association of Zoos and Aquariums (EAZA), British and Irish Association of Zoos and Aquariums (BIAZA), Association of Zoos and Aquariums (AZA), and Zoo and Aquarium Association (ZAA).

### 3.2 Roles of participants

A purposive sampling method was used to indicate the intended participant profile based on their role in the organisation. Each participating organisation received a letter with a link to the survey and a list of the identified roles for the purpose of this study. While we recognise a wide variety of roles in ZOAQs, for the purpose of this research, we focused on those directly involved or in management positions pertaining to supporting excellence in animal care and wellbeing. Individual participants were recruited across the following specific roles: Junior animal caregiver (Junior), Senior animal caregiver (Senior), Curator, Veterinarian, Veterinary professional (e.g., veterinary nurse), Animal welfare scientist/coordinator (AWS), CEO, and Other (e.g., nutritionists, registrars).

### 3.3 Sample size determination

Owing to the originality of this research and the lack of data on the effect size, it was not possible to perform a power calculation to determine the required sample size. Our proposed sample size of 250–300 participants was based on the need to obtain an adequate representation across a range of job/career-level positions and capture the variability across several ZOAQs worldwide. Previous research on laboratory animal technicians [(56), *n* = 90], animal care workers [(57), *n* = 139], and veterinary teams [(58), *n* = 274] also found this size sufficient to generate statistically meaningful results. We requested approximately 25 individual staff members in different positions per facility to complete the online survey.

The sample size target for the interviews was established at a minimum of 25, approximately 10% of the desired sample size, to reach saturation, loosely defined as information redundancy by Lincoln and Guba (59). However, recent developments in qualitative research methods suggest that a sample cannot be determined prior to data collection, or (at least some) data analysis (60). The data could be made up of a larger number of thinner (compared to broader sections) or shorter individual items, text lifted from individual interviews (61). Following Clarke et al. (62), the first author (SB) evaluated the available data during the interview process to assess the quality, richness, and diversity of meaning to determine when enough interviews were collected.

A total of 483 participants attempted the survey, and 442 completed at least half of the survey, with an average completion rate of 91.50%. Of 442 participants, 415 completed the survey. If more than half of the survey was completed, data were included in the analyses.

### 3.4 Survey design

Any identifying information obtained from the survey or follow-up interviews was removed to anonymise the participants’ profiles. A *Prefer not to say* option was added for all questions discussed in this article. The survey’s demographic questions included job position, gender, age, years working in the field, and highest educational level (see Supplementary Table 1).

Life satisfaction was assessed using the validated Satisfaction with Life Scale (SWLS) (22) which is a standardised measure to assess life satisfaction. The 5-item 7-point Likert scale assesses global cognitive judgements and does not measure positive or negative affect (63). Participants indicated how much they disagreed or agreed with each of the five items using an adapted version of this research survey on a 5-point scale (from a 7-point scale), ranging from 1 (strongly disagree) to 5 (strongly agree). This adaptation was implemented to keep the response options consistent across all Likert-scale questions within the survey. This is a valid and reliable scale (22), and the adapted version used in our survey had a Cronbach’s alpha of 0.89 [0.87 in (22)]. The total score, adding all item scores, was calculated and used in the analysis.

General self-efficacy was assessed using the validated PROMIS® Item Bank v.1.0—General Self-Efficacy, April 2020 version, (64) which has a 10-item scale and responds on a five-point Likert scale. Participants indicated how confident they felt with each of the 10 items using a 5-point scale that ranges from 1 = I am not at all confident to 5 = I am very confident. This is a valid and reliable scale (65), and the Cronbach’s alpha in our sample was 0.93. The total score, adding all item scores, was calculated and used in the analysis.

The previously validated single-item for individual job satisfaction question, *On the whole*, *how satisfied are you with your job?* (26) was assessed using a 5-point Likert scale (from a 7-point scale), ranging from 1 (strongly disagree) to 5 (strongly agree).

The organisational job satisfaction questions were inspired by, and adapted from, Boivin and Markert (27). It included 5 of the 6 original questions, all of which were positively worded (see Supplementary materials for all survey questions). The questions were assessed using a 5-point Likert scale (from a 6-point scale), ranging from 1 (strongly disagree) to 5 (strongly agree). The Cronbach’s alpha was 0.93, and we subsequently calculated the mean score for this variable to be used in analyses.

The five self-care categories were assessed using an adapted version of Butler’s (66) self-care assessment scale, originally published in Transforming the pain: A workbook on vicarious traumatization (67). The categories included physical [10 items, e.g., *exercised consistently (3–4 times a week)*], emotional (12 items, e.g., *allowed myself to accept time*, *help*, *advice*, *etc*. *from others*), spiritual (10 items, e.g., *cherished my own optimism and hope*), intellectual (six items, e.g., *improved my ability to say no when I want to*), and work-place self-care (8 items, e.g., *took time to connect with coworkers*). The questions were assessed using a 6-point Likert scale ranging from *never thought about that* (modified from *it never occurred to me*), *never*, to *always*. Cronbach’s alphas for each of the self-care categories in our sample were: physical 0.75, emotional 0.86, spiritual 0.84, intellectual 0.78, and work-place, 0.84, which mirror similar findings in the study with clinical nurses by Lee and Joo (68), using the full scale, as reported by Saakvitne and Pearlman (67). Given the inconsistency in item numbers across all categories, we subsequently calculated the mean score for each category to enable easy comparisons across categories. All survey questions not explicitly reported in this article are available for review in the Supplementary materials.

### 3.5 Statistical analysis

The following statistical analyses were conducted using SPSS version 28.0.0.0 (190). If more than half of the survey was completed, data were included in the analyses. Descriptive analysis (frequencies, means/median, and standard deviation) was used for all demographics, including gender, age, job position, time in the field (henceforth *Experience*), education level (henceforth *Education*), and how they perceived their job. Reliability analyses were conducted by calculating Cronbach’s alpha for the total life satisfaction, organisational job satisfaction, self-efficacy, and all self-care categories. Two contingency tables were created using Crosstab to analyse the relationship between job position and two different questions related to work-life and personal-life balance and integration. A One Way ANOVA and Cross tabs were conducted to evaluate the relationship between the two job satisfactions and calling. The Shapiro–Wilk Test for Normality (W) was conducted for all variables to understand their distributions. The distribution of total self-efficacy was significantly different from the normal distribution (W = 0.965, *p* < 0.001), without skew. The distribution of total life satisfaction was significantly different from the normal distribution (W = 0.974, *p* < 0.001), without skew. The distribution of organisational job satisfaction (Q18) was significantly different from the normal distribution (W = 0.957, *p* < 0.001). Except for the mean physical self-care (W = 0.995, 0.221), mean spiritual self-care (W = 0.997, 0.708), and mean workplace self-care (W = 0.995, 0.194), none of the other variables were normally distributed. The distribution of mean emotional self-care was significantly different from the normal distribution (W = 0.991, 0.014), without skew. The distribution of mean intellectual self-care was significantly different from the normal distribution (W = 0.992, 0.019), without skew. As most variables were not normally distributed, we used a two-tailed Spearman correlation to test the three hypotheses. A moderate positive relationship was found between individual job satisfaction and organisational job satisfaction (see Supplementary Table 2) and in the further analysis organisational job satisfaction was used in the GLM as this 5-item question was more robustly assessed. The Generalized Linear Models were conducted in R.

Three Generalised Linear Models (GLMs) were employed to identify significant predictors of the response variables: organisational job satisfaction, life satisfaction, and self-efficacy. These response variables were continuous and exhibited positive skewness. Therefore, all GLMs utilised a Gamma distribution with a log link function. The initial set of predictor variables considered for each model included demographic factors such as age, *Experience*, and *Education* as well as self-care dimensions such as Mean Physical Self-Care, Mean Emotional Self-Care, Mean Workplace Professional Self-Care, Mean Spiritual Self-Care, and Mean Intellectual Self-Care. Prior to model selection, multicollinearity among predictors in the full model for each outcome was assessed using Variance Inflation Factors (VIFs) to detect multicollinearity in multiple linear regression models (69). Job position and gender were removed from the analysis because of their high multicollinearity but are discussed in more detail in other sections of the paper.

For each response variable, a model refinement process was conducted using backward stepwise selection based on the Akaike Information Criterion (AIC). This approach aimed to identify the most parsimonious set of predictors of the respective outcomes. Model diagnostics were performed using the performance package (70). The fit of the final selected model for each outcome was compared to a null (intercept-only) model using a likelihood ratio test. Nagelkerke’s pseudo R^2^ was calculated as an estimate of the variance explained by each final model. Parameter estimates (coefficients), standard errors (SE), *p*-values, and 95% confidence intervals (CIs) were reported for the predictors retained in each final model, and the results were organised using the broom package (71). Statistical significance was set at an alpha level of *p* < 0.05.

All GLM analyses were conducted using R version 4.3.3 (72). The specific R packages utilised included dplyr for data (73), car for VIF calculation (74), broom for tidying model outputs (71), and performance for model diagnostics (70).

To investigate the differences in self-care engagement across domains, we calculated the deviation of the individual item means from the grand mean of the respective self-care domain. Responses were recorded on a 6-point Likert scale ranging from 1 (never thought about that) to 6 (always). Only valid responses were included in the analysis; therefore, options such as *Prefer not to say* or *Not applicable* were coded as missing values and excluded from all the mean calculations.

The grand mean (past studies) was computed by averaging the responses across all the items and participants for each domain. Subsequently, for each item within a domain, the mean score was compared to the corresponding grand mean, allowing for the identification of items where self-care engagement was relatively higher or lower. These deviations were visualised using bar charts to highlight the patterns of variation. Statistical significance of the deviations was assessed using one-sample Wilcoxon signed-rank tests. To account for multiple comparisons, the Bonferroni correction was employed, setting the significance threshold at an alpha level of *p* < 0.05.

### 3.6 Interview design

Semi-structured interviews were conducted with 39 participants (m = 20, *f* = 19) who completed the survey and signed up for follow-up online interviews to gain more insight into the lived experiences and perceptions of zoo professionals in diverse roles. Interviews were, on average, 1 h in duration, conducted, and recorded using Microsoft Teams at the University of Stirling. Key questions included for example, *Share how you feel supported in your job by your organisation* and *Share how you feel supported at work to care for your own wellbeing*. All transcripts were copied and anonymised and each participant was assigned a unique identification number. Any identifying details were cleared from each transcript. Best practices of thematic analysis (75), including data familiarisation, pattern identification, and coding, were used. All anonymous interviews were coded using NVIVO 1.7.1 for two rounds by SB, and quotes were collated in an Excel document. After two rounds, all codes were reviewed by SB and LC and further collated into larger themes, after which all interviews were reviewed a third time. All questions are available in the Supplementary materials.

## 4 Results

### 4.1 Participant demographics and descriptive results

All participants were from 23 ZOAQs. Most participants were female (*n* = 296), the most frequently reported job position was Senior, and most were in the age bracket 31–35 years. Most participants reported having 6–10 years of *Experience* working in the animal care and welfare domain, and a BSc was the most reported degree for the question regarding the highest level of *Education*. The distribution for age was significantly different from the normal distribution (W(410) = 0.931, *p* < 0.001) and skewed towards people younger than 40. The distribution of job positions was significantly different from the normal distribution (W(410) = 0.760, *p* < 0.001) and skewed towards Junior and Senior professionals. The distribution of time spent working in the field (*Experience)* was significantly different from the normal distribution (W(433) = 0.909, *p* < 0.001) and skewed towards younger professionals, with most participants being 1–10 years on the job. The distribution of *Education* was significantly different from the normal distribution (W(410) = 0.850, *p* < 0.001), with most participants having some university-level training. Detailed demographics are available in the Supplementary Table 1.

**Figure 1:**
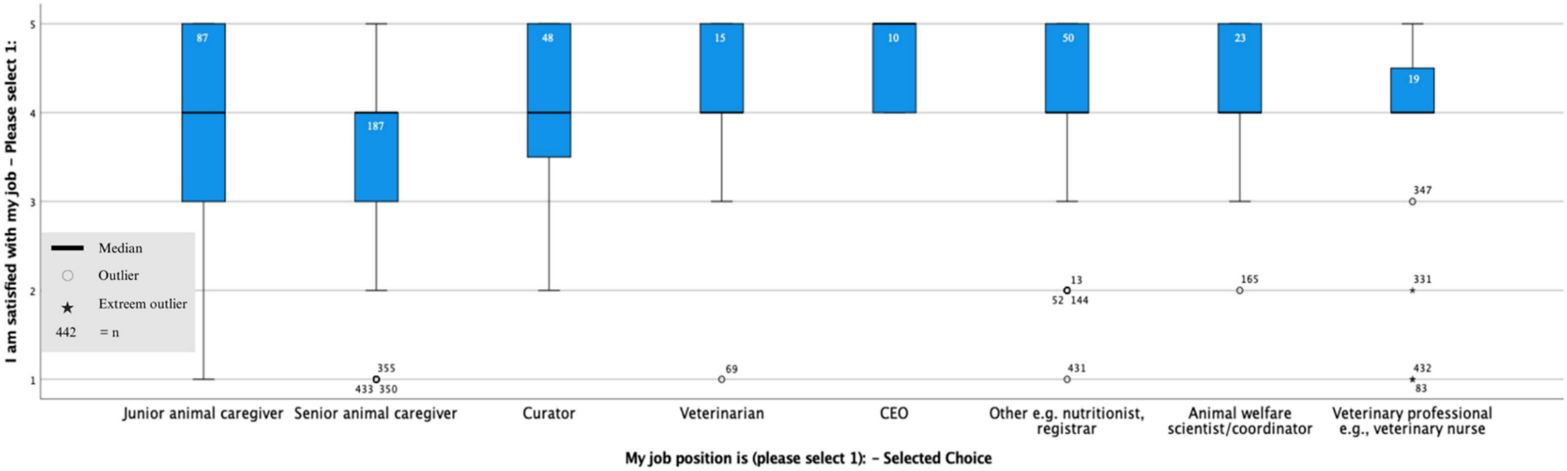
Boxplot representing the variability of responses to the question *I am satisfied with my job* for different job roles, showing the largest variability for juniors, with CEOs reporting the highest median job satisfaction scores. This boxplot includes outliers and extreme outliers due to the non-normal distribution of the data.

For the single question, *I am satisfied with my job*, most participants responded somewhat agree (44.3%), followed by strongly agree (27.4%), neither agree nor disagree (11.1%), somewhat disagree (11.1%), and strongly disagree (6.1%). The distribution of individual job satisfaction was significantly different from the normal distribution (W(442) = 0.831, *p* < 0.001), skewed to those somewhat agreeing to be satisfied with their jobs. The box plot in Figure 1 shows the largest variability for Juniors, with CEOs reporting the highest median job satisfaction scores.

For the question on ‘I consider my current job to be’ (27), most participants considered their job to be a calling/passion (*n* = 259), followed by a career to remain in the field (*n* = 153). A minority considered their job “a job I have some interest in doing” (*n* = 26), and only 1 participant indicted that it was just a way to make money. Three people selected the *Prefer not to say* option. The distribution for the variable considering their job as a calling was significantly different from the normal distribution (W = 0.974, *p* < 0.001), skewed to the job being considered a calling. No relationship was found between job calling and organisational job satisfaction in the correlation analysis (r_s_ = −0.18, *p* = 0.710), with an ANOVA confirming the absence of a relationship (*F*(3, 433) = 1.652, *p* = 0.177). However, a relationship was found between job calling and individual job satisfaction in the correlation analysis (r_s_ = 0.621, *p* < 0.001) and an ANOVA confirmed this relationship: *F*(3, 435) = 5.313, *p* < 0.001. Please see Supplementary Table 2 for the cross tab providing more details on the found association.

Concerning the question related to work-life (WL) and personal life (PL) balance, participants across all job positions reported that work-life and personal-life balance was balanced more towards work, with differences between job positions (see Supplementary Table 2). Veterinarians and veterinary professionals were the only job positions that did not report WL and PL integration to be balanced towards life. Juniors, Curators, AWSs, and Others reported WL and PL more frequently than WL and PL balanced towards life. Concerning WL and PL integration, participants across all job positions reported that their integration was largely satisfactory, with additional differences between job positions (see Table 1).

**Table 1:** Contingency table comparing job position and perception of work-life (WL) and personal-life (PL) balance.

Job position	My WL and PL integration is balanced more towards work		My WL and PL integration is balanced more towards life		My WL and PL integration is mostly even	
CEO	7	2.5%	1	4.2%	2	1.5%
Other	29	10.3%	5	20.8%	16	12.1%
Veterinarian	10	3.6%	0	0.0%	5	3.8%
Veterinary professional	12	4.3%	0	0.0%	7	5.3%
Animal welfare scientist/coordinator	13	4.6%	2	8.3%	8	6.1%
Curator	32	11.4%	3	12.5%	13	9.8%
Senior animal caregiver	111	39.5%	12	50%	62	47.0%
Junior animal caregiver	67	23.8%	1	4.2%	19	14.4%
Prefer not to say	5	1.1%	5	1.1%	5	1.1%

This table displays the crosstab results for the question related to work-life (WL) and personal life (PL) balance for different job positions (n = 437). Most participants indicated that integration is more balanced towards work. Pearson Chi-Square = 14.96 df = 14, *p* = 0.381.

### 4.2 Descriptives and correlations of key variables

**Figure 2:**
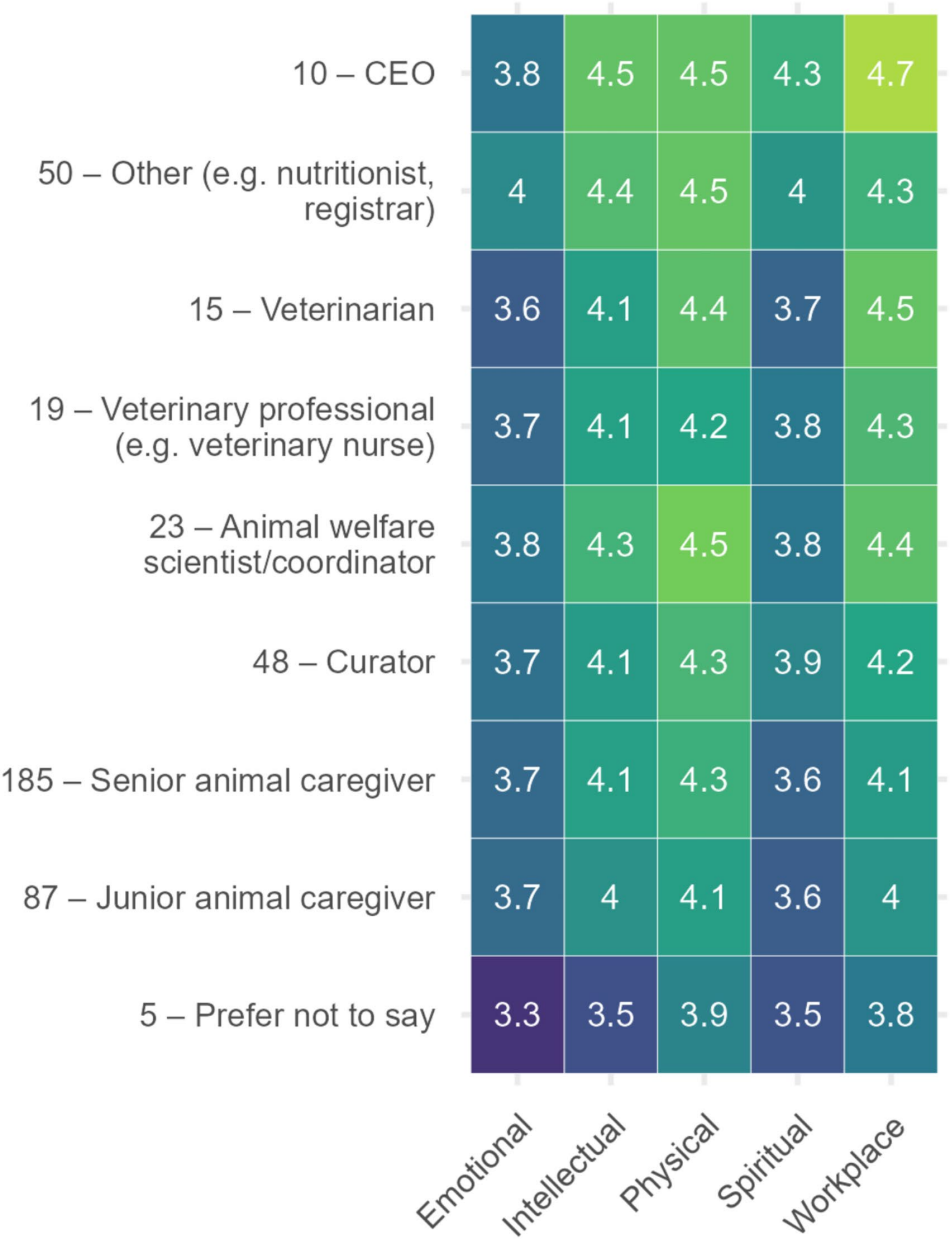
This heatmap illustrates the mean self-care domain scores for each self-care category for each job position (with sample n provided before job position), with the scale ranging from 3.0**–**5.0. The brighter the square, the more the person in that job role engaged in the corresponding self-care, e.g., high engagement of CEOs in workplace self-care, while the lowest engagement across all job roles in emotional self-care, are those in the *Prefer not to say* category.

The heatmap in Figure 2 illustrates the mean self-care domain scores for each job position, with a scale ranging from 3.0 to 5.0. The brighter the square, the more the person in that job role engaged in the corresponding self-care, e.g., high engagement of CEOs in workplace self-care, while the lowest engagement across all job roles in emotional self-care, are those in the *Prefer not to say* category.

### 4.3 Correlations

Spearman correlation analysis showed a very weak positive significant relationship between self-efficacy and levels of organisational job satisfaction (*r* = 0.194, *p* < 0.001), a weak positive significant relationship between self-efficacy and levels of satisfaction with life (*r* = 0.307, *p* < 0.001), and a moderate positive relationship between organisational job satisfaction and levels of satisfaction with life (*r* = 0.439, *p* < 0.001). A very weak positive significant relationship between self-efficacy and levels of individual job satisfaction (*r* = 0.161, *p* < 0.001), and a moderate positive relationship between individual job satisfaction and levels of satisfaction with life (*r* = 0.478, *p* < 0.001) were also found (Table 2).

**Table 2:** Spearman rho correlation results table for satisfaction with life, organisational job satisfaction, self-efficacy, and all types of self-care.

Variables	N	1	2	3	4	5	6	7	8	9
1 Mean organisational job satisfaction	440	1.00	0.44**	0.19**	0.29**	0.26**	0.26**	0.30**	0.46**	0.62**
2 Total life satisfaction	426		1.00	0.30**	0.42**	0.39**	0.42**	0.44**	0.46**	0.47**
3 Total self-efficacy	426			1.00	0.19**	0.35**	0.21**	0.26**	0.23**	0.16**
4 Mean emotional self-care	437				1.00	0.65**	0.55**	0.62**	0.57**	0.25**
5 Mean intellectual self-care	441					1.00	0.56**	0.52**	0.51**	0.26**
6 Mean physical self-care	442						1.00	0.45**	0.53**	0.23**
7 Mean spiritual self-care	434							1.00	0.47**	0.27**
8 Mean workplace professional self-care	433								1.00	0.46**
9 Single question individual job satisfaction	442									1.00

This table illustrates that all Spearman rho correlations between Satisfaction with Life, Individual and Organisational job Satisfaction, Self-Efficacy, and All Types of Self-care are significantly positively associated. These findings resulted in further exploration of the data using Generalised Linear Models. Correlation is significant at the 0.01 level (2-tailed).

### 4.4 GLM

#### 4.4.1 Model fit and retained predictors

**Figure 3:**
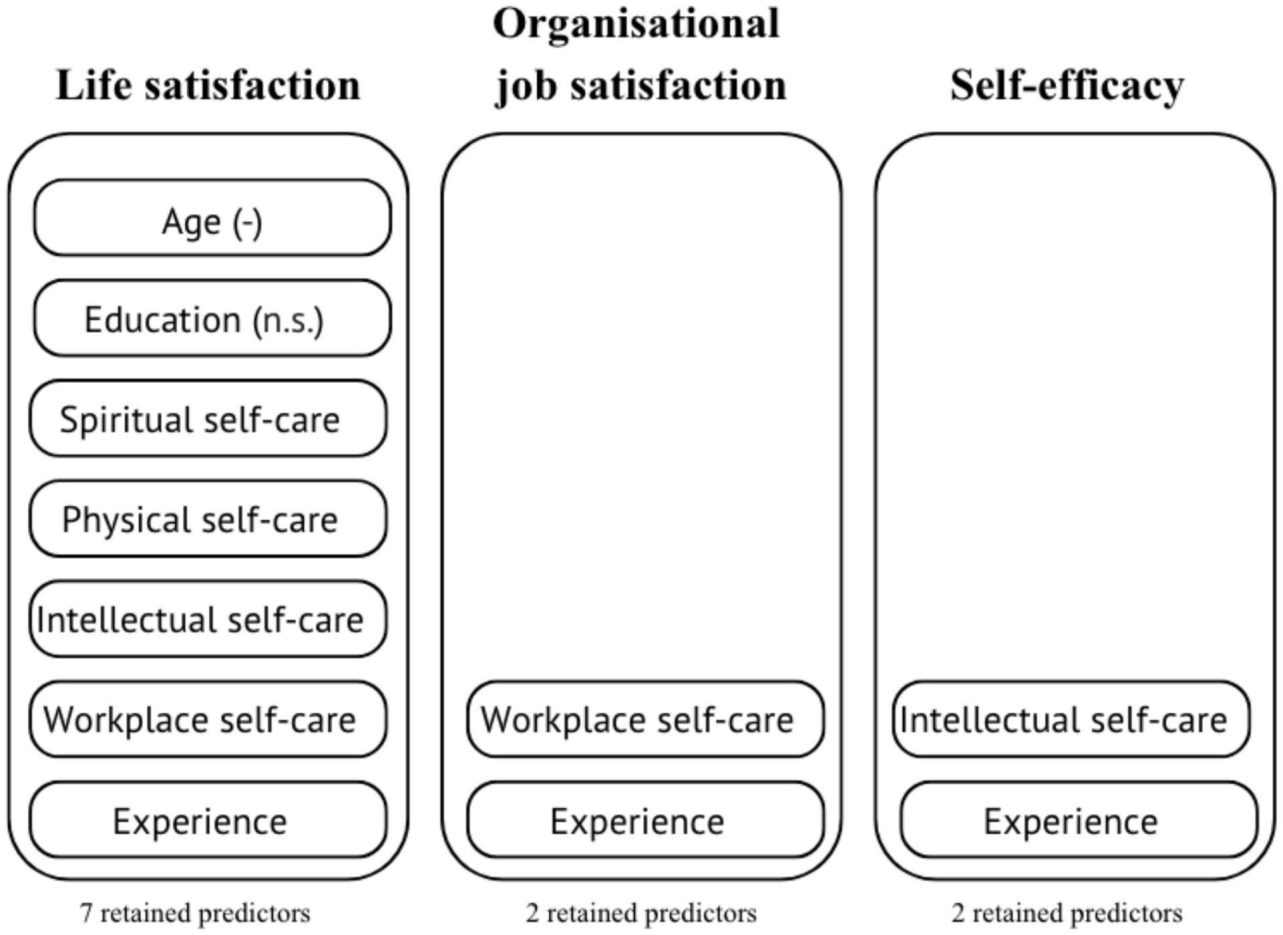
This figure illustrates the retained predictors of three GLMs: Life satisfaction, organisational job satisfaction, and self-efficacy. *Experienc*e was retained as a positive predictor across all three variables, while Workplace professional self-care was retained only for life satisfaction and organisational job satisfaction (positive association). Emotional, physical, and spiritual self-care (positive association), along with *Education* (no significant association) and age (negative association) were retained for life satisfaction.

The final GLMs for organisational job satisfaction, life satisfaction, and self-efficacy all provided a significantly better fit to the data than their respective null models (*p* < 0.001 for all likelihood ratio tests). Table 3 illustrates the parameter estimates standard errors, *p*-values, and 95% confidence intervals for the predictors retained in each final model after the backward stepwise selection. Retained predictors of life satisfaction, organisational job satisfaction, and self-efficacy are illustrated in Figure 3.

**Table 3:** Summary of the parameter estimates, standard errors, *p*-values, and 95% confidence intervals for the predictors retained in each final model after the backward stepwise selection.

Response variable	Predictor	Estimate	Std. error	*p*-value	95% CI (Low)	95% CI (High)
Job satisfaction (R^2^ = 0.178)	(Intercept)	0.1146	0.1059	0.280	−0.0952	0.3257
	Experience	0.0190	0.0098	0.053	0.0001	0.0380
	Workplace professional selfcare	0.2266	0.0241	<0.001	0.1787	0.2744
Life satisfaction (R^2^ = 0.287)	(Intercept)	1.7243	0.1059	<0.001	1.5167	1.9322
	Age	−0.0138	0.0073	0.058	−0.0280	0.0003
	Experience	0.0242	0.0088	0.006	0.0071	0.0414
	Education level	0.0241	0.0147	0.102	−0.0052	0.0533
	Physical self-care	0.0736	0.0242	0.003	0.0264	0.1208
	Emotional self-care	0.0529	0.0260	0.042	0.0023	0.1036
	Workplace professional self- care	0.0793	0.0218	0.0003	0.0365	0.1221
	Spiritual-selfcare	0.0527	0.0216	0.015	0.0107	0.0947
Self-efficacy (R^2^ = 0.130)	(Intercept)	3.2433	0.0510	<0.001	3.1442	3.3428
	Experience	0.0169	0.0049	0.0006	0.0073	0.0266
	Intellectual self-care	0.0811	0.0114	<0.001	0.0589	0.1032

Experience is retained for organisational job satisfaction, life satisfaction and self-efficacy, Workplace professional self-care is retained for both organisational job satisfaction and life satisfaction, and Intellectual self-care is retained only for self-efficacy. The other types of self-care (physical, emotional, workplace and spiritual) were retained for life satisfaction as well as age (negative) and *Education* (despite not being significant).

#### 4.4.2 Organisational job satisfaction

The final GLM for mean organisational job satisfaction explained approximately 17.8% of the variance (Nagelkerke’s R^2^ = 0.178). After backward stepwise selection, two predictors were retained in the model. *Experience* showed a positive association (Estimate = 0.0190, SE = 0.0098, *p* = 0.053), suggesting that individuals with more time in the field tended to report higher organisational job satisfaction. Workplace professional self-care.

Age, *Education*, mean Physical self-care, mean Emotional self-care, mean Spiritual self-care, and mean Intellectual self-care were excluded during model selection.

#### 4.4.3 Deviation from the mean

**Figure 4:**
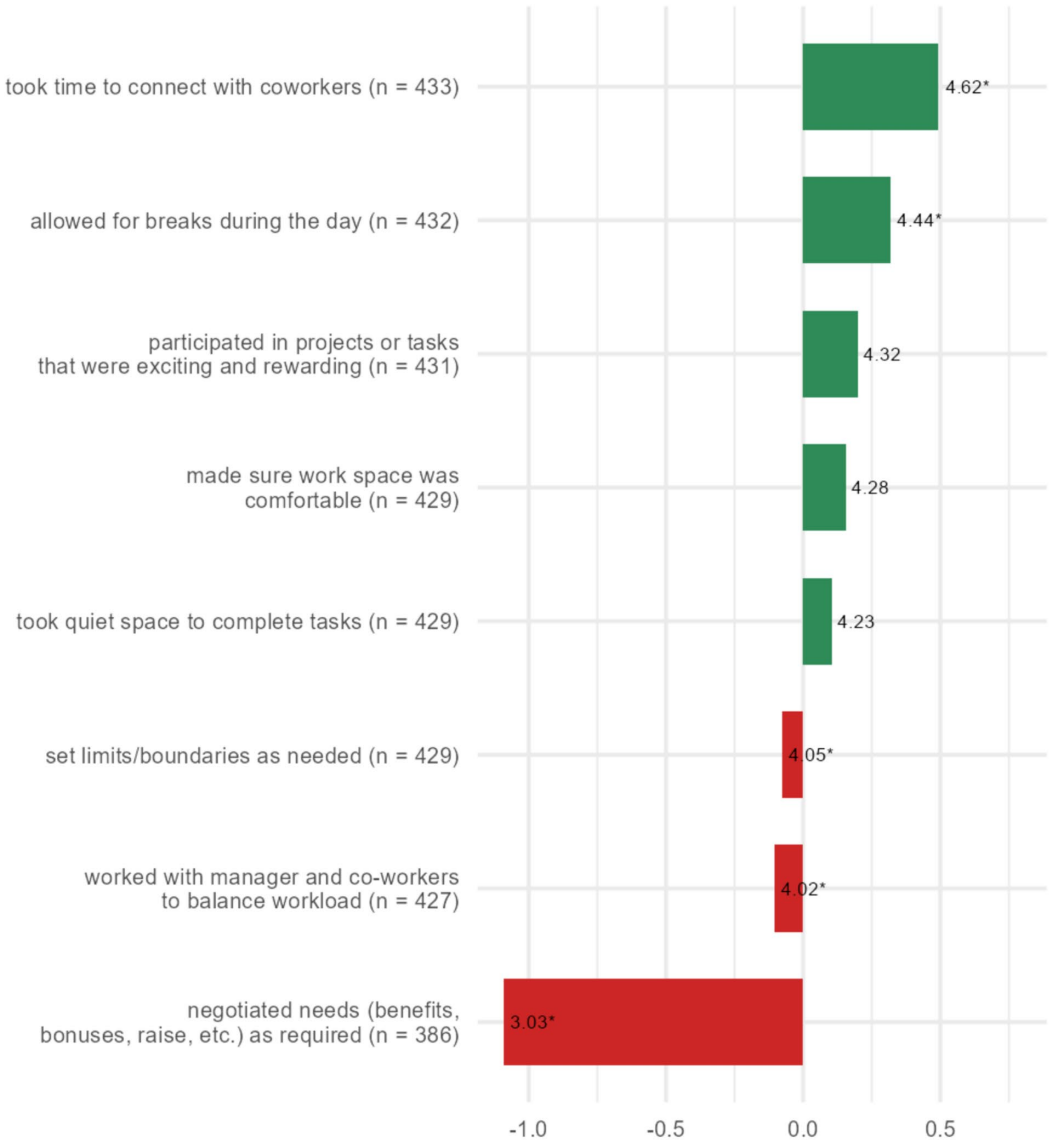
This figure illustrates the deviation from the mean for each individual item included in the Workplace self-care domain, and in which of the self-care item(s) a participant was more likely to engage. Likert scale mean score (1–6). *p < 0.05 (Bonferroni-adjusted Wilcoxon test).

First, Spearman’s correlation was used to measure the strength and association of different self-care variables. All mean self-care variables were significantly positively correlated (Table 4). All job positions and individual self-care items were combined to explore which individuals were more likely to engage in each self-care domain. For example, people across all job positions were more likely to take time to connect with coworkers and take breaks during the day, then to set limits/boundaries as needed or work with a manager or co-worker to balance workload. Figure 4 illustrates the deviation from the mean for each item in the Workplace self-care domain. Supplementary Figures 5–8 illustrates the deviation from the mean for each of the individual items included in all other self-care domains; Physical, Emotional, Spiritual, and Intellectual.

**Table 4:** Contingency table comparing job position and perception of work-life (WL) and personal-life (PL) integration.

Job position	My WL and PL integration is problematic		My WL and PL integration is satisfactory		My WL and PL integration is harmonious	
CEO	3	2.0%	6	2.5%	1	2.1%
Other	14	9.2%	31	13.0%	5	10.6%
Veterinarian	4	2.6%	9	3.8%	2	4.3%
Veterinary professional	5	3.3%	11	4.6%	3	6.4%
Animal welfare scientist/coordinator	5	3.3%	14	5.9%	4	8.5%
Curator	12	7.9%	30	12.6%	6	12.8%
Senior animal caregiver	67	44.1%	97	40.8%	21	44.7%
Junior animal caregiver	42	27.6%	40	16.8%	5	10.6%
Prefer not to say	5	1.1%	5	1.1%	5	1.1%

This table displays the crosstab results for questions related to work-life (WL) and personal life (PL) integration for different job positions (*n* = 437). Most participants across all job positions reported their integration to be largely satisfactory. Pearson Chi-Square = 14.988, df = 14, *p* = 0.379.

#### 4.4.4 Thematic analysis

While the interviews covered a wide variety of topics and hence resulted in a large number of themes, the ones relevant to the focus of this article are the following four themes: *Challenges to and in the zoo community* which included reoccurring issues and obstacles to change; *life stages of the profession*, which included perceptions of position and life lessons learned; *perceptions of the field and their job* which included their perceptions and lived experiences at work; and *self-care work-life and personal life balance* which included self-care at work and self-care outside of work. Quotes were selected based on their relevance to the main findings and reflecting the demographic variability of the sample.

The theme, *Challenges to and in the zoo community* included topics such as recurring issues, obstacles to change, charity status, and private versus public ownership. A charity status allows donations and potential volunteers, but some of the reoccurring issues or obstacles to change, such as requests for animal enrichment or training budgets, might be limited due to reliance on charity funds.

The relevance of our park, it is not strong in terms of conservation. It's still very much based on like a tourism attraction and entertainment venue kind of concept and we're always like very guest centred (161, Senior).

We take a survey every year to help…for two years, it's been overwhelmingly negative…at staff meetings, they recognise that it's negative and to fix this, but it’s like they're trying to guess. We don't see a lot of actions (154, Senior)

Public ownership may cause challenges with decentralised organisations, where the animal team may be disconnected from other teams and support which may be located outside the zoo grounds; conversely, people may have more support through a union or job protection. Private ownership may provide more flexibility and opportunities, as well as less bureaucracy, but may increase favouritism for family and friends and less support and protection in challenging times.

The private owner has the ultimate say…people in terms of being let go and the animal side of things. And unfortunately, in both of those cases, there was a lot of nepotism and like, family would basically get privileges, that even if they didn't deserve those roles or responsibilities (198, Senior)

The theme *Life stages of the profession* include perceptions of the position and lessons learned through experience. Aspects of education, opportunities, and growth through different career stages, such as going on courses, conferences, or training on the job (or the lack of all or some of these). Confidence in ability and skill, how they assist in navigating differences between approaches and values of different staff members. Finding integration of work with other aspects of life such as more reasonable workloads, working conditions, and pay as they levelled up, or changes made due to internal pressures from staff in the field, were all brought up in this theme. One interviewee reported “I’ve been through the growing pains. I think I’m at a point where I have found a good balance, but it took a long time” (156, Senior).

It is a lot different from when I started on minimum wage. Especially after COVID a lot of zoos changed because people reevaluated what's important. Do I need to work? Can I find something that fits my needs better, even if it's fewer hours, giving me flexibility to take care of things? I think that benefited people. I have seen these changes in salary that have taken place over the past few years (245, Curator)

There's the old guard, this is how we've always done it kind of people, but I feel a lot of people my age, we know that change can't happen without everyone contributing (130, Senior)

The theme *Perceptions of the field and their job* included topics highlighting their perceptions and lived experiences at work and was kept broad to include a diverse set of responses. This could include lived changes in the field across time, for example, more attention to animal and staff wellbeing over the last decades or a continued lack of budget, time, and staff. Aspects contributing to wellbeing were widely covered in this theme, such as having a support and social system outside the zoo context, providing a living wage, or the challenges of low pay and high living costs (in particular in cities), support such as continuous development opportunities or staff appreciation days, high turnover, lack of respect, and care.

They really couldn't care less if I am sick, or if I am crying in the toilet, or if I'm suffering or in pain. As long as the exhibit is good and the birds fly out, I'm doing a good job. It's not like they're going to tell you directly. We were on minimum staff, and one needed to go home. We were only two people, and needed at least three people, which means we are really going to be late and struggling. They will not pay for that because at X never paid me for any extra work that I did (56, Junior)

I don't think anyone can have a good sense of wellbeing if they're just hanging out with zoo colleagues all the time and talking about work, because that will quite often, even if you're positive, turn into negative, especially if you're in a role, we're sitting there going, why like, why didn't management do that? (378, AWS)

I feel it would be very near impossible to start one [*a career*] unless you are young or have a low budget…and like where I've been, they've not the best at keeping staff. They've lost quite a few in recent years and have never looked at themselves. They always look at people other than their issues. We had at least one year where 12 left…and it's one of those things, you kind of get it. I don't even know if one should start because of just the fear of what could happen because you see all your friends leave, they don't bat an eye, so you go okay, well then, what's the point? (114, Senior)

*Self-care work-life and personal life balance* include self-care at work and self-care outside of work. This could include the effects of work on wellbeing, such as good work relationships, getting out on time most of the time, feeling that one is contributing to shared goals, and balancing work life with self-care at home. A large part of this theme consisted of discussions on applying professional workplace self-care such as connecting with coworkers and engaging in rewarding tasks. Self-care activities outside of the work context, focused mostly on seeking social support from friends, family, and local community, or the lack of support because of being in a remote area. Lastly, this theme also included challenges faced between caring for oneself and caring for others, such as caring for the family, team members at work, and caring for oneself when overworked or overwhelmed (from home to work, and vice versa).

I am trying to take better care of myself. I need to get better at eating healthier, just be healthier (143, Curator)

If things aren't going well, it starts to affect your work, your life, and everything that you bring into work as much as you try not to. When things going on at work cause frustrations, feeling the work stresses and being mentally exhausted, I wasn't my usual working self and wasn't sharp (140, Senior)

I'm very lucky, I have a great family at home. My boyfriend is very supportive. My family just thinks I have the coolest job ever. They're always happy to hear my stories, talk about, and decompress (143, Senior)

I try to exercise regularly, just by messing with friends who are not in the zoo. I remind myself every day, okay, that's what happened, you can make something of it. You can sit around and mope, or decide that's what happened and now we have to move forward, learn from it and go on (400, Veterinarian)

## 5 Discussion

This research sheds light on how zoo professionals with different roles in ZOAQs perceive their overall life satisfaction, individual and organisational job satisfaction, and self-efficacy and which types of self-care contribute to these variables. Most participants (71%) agreed or strongly agreed to the statement *I am satisfied with my job*, which was particularly true for those who consider their job to be a calling. The correlation between self-efficacy and organisational job satisfaction was found to be very weakly positive and significant, likely due to the complexity and multifaceted nature of this relationship. Additionally, a weak positive significant correlation was observed between self-efficacy and life satisfaction, while a moderate positive relationship existed between organisational job satisfaction and life satisfaction. Most participants reported being satisfied with their jobs, although there were notable differences in satisfaction levels across different job roles. The deviations from the mean for all the self-care categories were further explored, and across individual items, to further understand the types of self-care that individuals in this study were more likely to engage in, or not. The GLM analyses revealed that only one factor, *Experience*, contributed to all independent variables, suggesting that participants with more time in the field reported higher total life satisfaction, organisational job satisfaction, and levels of total self-efficacy. For total life satisfaction, four self-care factors (physical, emotional, spiritual, and workplace) were significant positive contributors. Workplace self-care positively contributed to organisational job satisfaction, whereas intellectual self-care contributed positively to self-efficacy.

### 5.1 Experience and organisational job satisfaction

*Experience* (time in the field) and Workplace self-care (e.g., *connecting with coworkers*) were the two predictors retained in the final GLM for organisational job satisfaction. This aligns with previous studies in other fields, such as hospital workers, reporting that the job itself, the nature of the work, satisfaction with superiors, and cooperation with colleagues contribute to general job satisfaction (76), while these participants reported low satisfaction with salary, rewards, and opportunities for promotion. Experience can be built through vocational and academic education as well as outside the formal classroom setting through internships, volunteer work, and building professional networks (77, 78). The significant role of *Experience* in the GLM links with the theme identified *Life stages of the profession* in the thematic analysis. As people enter the workforce, they build experiences by practising new skills through watching peers, in-house training, activities, mentors, continuing professional education, and practical opportunities (1). This is illustrated by this quote: *“And I was really lucky because*, *you know*, *we had a big team with lots of different expertise*. *It wasn’t just me trying to figure out everything” (192*, *Senior)*.

People also learn in their personal lives, from family, friends, and community, through hobbies, travelling, and other activities they enjoy. Especially in professions seen as a calling, like zoo professions, the boundaries between personal and work-based learning are more blurred, and zoo professionals are known to use their own time and money to travel to conferences, go on courses, or engage in work exchanges (personal experience Sabrina Brando). This also includes aspects such as spending time and personal money on education and activities which should be more supported from an organisational perspective, given the benefits they reap, as one interviewee stated, “I regularly buy enrichment or treats for the animals, … and pay for conferences as I only get my days off” (Senior, 126). Diverse capabilities, activities, and continued learning increases overall experience, and as illustrated in our findings for this group of participants, contributes to overall life satisfaction and self-efficacy (1, 79).

Organisational job satisfaction and self-efficacy have been shown to positively correlate with work engagement by providing a supportive work environment, sufficient job resources, positive feedback, and various opportunities for training (79). Despite the very weak significant positive correlation between organisational job satisfaction and self-efficacy, we indeed also found that workplace self-care substantially contributes to organisational job satisfaction. When looking at the individual items in the category of workplace self-care (see Figure 4), we can see that these participants were engaging significantly in: *took time to connect with coworkers* and *allowed for breaks during the day*. Conversely, these participants are significantly less engaging in *set limits/boundaries as needed, worked with manager and co-workers to balance workload,* or *negotiated needs (benefits, bonuses, raise, etc*.*) as required*. Considering participants’ experiences as illustrated in the thematic analysis and discussion of the demographics, we believe that some of the reasons for the lack of engagement with appropriate workplace self-care have to do with perceived working conditions and overall insufficient leadership and organisational culture to support staff. The need for improved communication and support was also identified in the theme *Challenges to an in the zoo community* in the thematic analysis and is illustrated by the following quote:

I feel the communication is a big one. I feel communication sucks every time. We have meetings and we talk and strategic plans about it. And nothing changes. Well, that was a lot of time we spent, and it's still the same (56, Junior)

### 5.2 Self-efficacy

As defined in the introduction, self-efficacy is the belief in one’s ability to successfully perform a specific task or achieve a desired outcome. Bandura (80) first introduced the concept of self-efficacy as a key component of social cognitive theory, emphasising its role in motivation, behaviour, and behavioural change. Bandura specifically noted that self-efficacy is not about having a general belief in oneself but rather a specific confidence in one’s capabilities in a given domain or situation. Relevant to the current context, individuals, particularly when not in leadership roles, do not have a significant influence on organisational decision making, processes, salary negotiations, workload, or support (81). However, individuals can control their day-to-day activities and interactions to a certain extent, such as connecting with coworkers or participating in rewarding tasks. These findings support previous findings that the job itself, the nature of the work, and connection with colleagues contribute to organisational job satisfaction and self-efficacy, as these fall under the control of the individual. This aligns with PWB, which encompasses a broader sense of flourishing - denoting good mental and physical health (18), and includes aspects like autonomy, personal growth and relationships, purpose in life, and environmental mastery (19).

Self-efficacy therefore reflects confidence in the ability to exert control over one’s own motivation, behaviour, and social environment, which may explain why Intellectual self-care and *Experience* were retained as positive predictors of self-efficacy in this study. Self-efficacy can be enhanced through mastery experiences (80), which involve tackling and succeeding in challenging tasks to build confidence (82), but also grows through different working experiences across a career through the building up of personal and professional resources. For example, research has shown that higher self-efficacy and personal resources for resilience in veterinary practice are related to higher resilience (83). Wood and Bandura (84) stated that “if people receive realistic encouragement, they will be more likely to exert greater effort and to become successful than if they are troubled by self-doubts,” which can also be referred to as verbal persuasion. This highlights how self-efficacy can be supported in both work and personal life environments and enforces learning processes and experiences across time. Indeed, a general survey of working professionals found that negative work events hamper daily goal attainment and reduce employees’ daily self-efficacy (85). Studies have consistently shown that perceived organisational support positively influences self-efficacy (86, 87). The importance of organisational support for self-efficacy was also demonstrated in our findings when looking at the individual items in the category of intellectual self-care (see Supplementary Figure 8). As evident from the deviation from the mean results on intellectual self-care, participants were engaged significantly more in behaviours like *wore clothes I like*, while participants were significantly less engaging in *improved my ability to say no when I want to, said no to people crossing my boundaries (*e.g.*, not working another shift*, or *tried new things)*. We recommend the need to nurture organisational cultures, emphasising the importance of professional self-care. The need for an improved culture of care was identified in the theme *Perceptions of the field and their job* in the thematic analysis, as illustrated by the following quote:

We are in a tough industry, one that feels very old. We're unappreciated, get paid like debt, having to work overtime without any of those sort of benefits, or recognitions, we’re not appreciated, we’re not respected (256, Senior)

### 5.3 Life satisfaction

Understanding the contributions to life satisfaction is the most complex and nuanced of the three variables, as there are many aspects which affect overall life satisfaction (88–90). Different dimensions of wellbeing that contribute to overall life satisfaction are finance, environmental, social, and occupational (88, 91). The retention of the physical, emotional, spiritual, and workplace self-care as positive predictors and *Experience* reflects the complex nature of life satisfaction. This aligns with SWB, which focuses on the individual’s subjective evaluation of their life, including the cognitive component of satisfaction with life as a whole (21) and the balance of positive and negative emotions (22–24). These complexities were further reflected in the themes *of Self-care work-life and personal life balance*, which identified a difficult balance between caring for themselves, caring for others and work-related responsibilities or conditions. For example, when looking at the individual items of Emotional self-care (see Supplementary Figure 6), we see that participants are engaging significantly more *in Allowing for quality time with others who have a positive impact on my life*, which is also neatly illustrated by the quote:

My boyfriend is really great about stuff. I talked to my mom all the time. I said, I do have a therapist and she's super helpful with perspective and stuff. And I have friends who some of them used to be in the animal field. Some of them have never been in the animal field… I do have friendships with people that I work with too. But sometimes it's nice to have somebody who's completely not connected help validate you, which I think is great. (130, Senior)

When looking at the individual items of physical self-care (see Supplementary Figure 5), we see that participants are significantly less engaging in *Took time off when I got sick, Got 7–9 h of sleep per night,* or *Digitally unplugged 1 h before sleep* which suggests that the types of self-care that support good and sustainable wellbeing are not frequently engaged in. This may include irregular sleeping patterns, or not freeing time to engage in other activities promoting optimal wellbeing. One interviewee indeed stated:

I sleep a lot. I think many spend time sleeping when we're not at work. For a lot of our days off it will be like what do you do for of them? I slept. (161, Senior)

When looking at the individual items in Spiritual self-care (see Supplementary Figure 7), we can see that participants were significantly less engaged in *Participate in a community that shares my core values*, which is a curious finding. While this needs further exploration, this may suggest that many participants in this study did not view their workplace as a community that shared their values. One interviewee noted “*I do not get super motivated by our mission, but the everyday tasks so my team is wonderful*” (27, Junior).

As *Experience* is a key variable explaining self-efficacy, organisational job satisfaction, and life satisfaction, exploring the findings more in the context of the participant’s characteristics, including *Education*, job position, and age, is relevant to gaining a better understanding. Moreover, given the varied impact of self-care depending on the variables, there is a need to better understand whether the experiences of people in this field align with the desired and needed experiences which are meaningful and supportive in demanding professions. Many individual self-care items contribute to overall life satisfaction and Workplace self-care to self-efficacy and organisational job satisfaction. However, many self-care items, including Workplace self-care, were also significantly less engaged in by the participants in this study, similar as reported by Cassie and DuBose (49). This suggests that people may have missing awareness, support and training regarding the importance of overall self-care, and the need for both the organisation and individual acknowledge commitment and responsibility to self-care.

### 5.4 Participant profiles

Across different job positions, most participants were female. While not extensively studied in animal caregivers, it does reflect a gender distribution change seen in veterinary professions over the past decades (92, 93). Although gender was not extensively studied in this research and had to be removed in the GLM due to high multicollinearity, we stress the importance of hearing all voices, including but not limited to gender, which requires future research. This study highlights that a third were new professionals, with most participants being 6–10 years (22.2%) in the field, followed by those working only 1–5 years (19.7%). Less than 13.8% had more than 15 years. One interview in this study noted the following regarding age and gender:

We have a fairly young workforce in the keepers…a lot of women between 25 and 40…who go off and have babies, so we have lots of maternity leave and short-term absences, so there are a lot of people in acting positions [*temporary positions*] (399, Manager)

The reduced lack of *Experience* in ZOAQs has grown exponentially since COVID, when many staff members were unfortunately furloughed and laid off because of a lack of funding, as documented in many newspapers worldwide [Brookfield Zoo (94, 95); UK Zoos (96)]. Various impacts of COVID on animal wellbeing and conservation have been studied (97, 98), but few studies have looked at staff wellbeing in ZOAQs, and this paper adds to this evidence.

The zoo and aquarium fields have often seen turnover, or people thinking about leaving the field altogether for various reasons, including low pay, overwork, conflict with co-workers, inadequate support, and leadership (1). One interviewee said, “We lost 12 keepers in my two and a half years there. Just in my team, there’s a high turnover rate (70, Junior).” Another interviewee noted “If my wife wasn’t doing her current job, I would absolutely have to work two jobs (119, Senior).” Job demands, emotional and moral stress, staffing and scheduling, and inadequate compensation have been highlighted in other animal professions such as laboratory personnel (99), animal shelters (100), and animal workers in rescue and management (101). One interviewee noted, “I would like get rid of this like toxic overwork mentality, like it’s become something that everybody brags about (378, AWS).” An interviewee who has since left stated “It has got bad leadership, it has bad people in charge of it. If that changes, I think this place could get so much better” (114, Senior). Research has shown that zoo professionals who are satisfied with their jobs, feel respected, and experience low levels of work stress tend to have longer careers in ZOAQs (1).

Most participants who completed the survey were Seniors (41.9%), followed by Juniors (19.7%). Other animal-related professions such as curators and AWSs also contributed substantially to this research compared to the number of these positions represented in ZOAQs. However, the participation of those in leadership, as indicated by the self-described job position in the *Other* category, was considerably lower than expected. Only 10 participants in the *Other* category indicated being in a leadership position like Director. Furthermore, out of the 23 organisations participating, only 10 CEOs took the time to participate in this multi-level research to shed light on individual, team, leadership, and organisational aspects of human wellbeing. Only one participant indicated working in policy, risk, and governance, and no one from the Human Value Department (formerly known as Human Resources) participated in this research. We acknowledge that directors and CEOs are busy individuals, as are animal caregivers and other professionals throughout the organisation. The low participation of program directors and the CEOs may indicate a lack of urgency and acknowledgement of the importance of this topic in their organisation. One interviewee stated, “We have senior management which are executive directors, finance, and the CEO, and so on, and they have a life of their own dealing with the board and with the finances (361, Director).” Another interviewee noted “The HR department put a sticky note that said, nothing is wrong, everything is totally fine. And we are like, that’s the problem, you guys have no idea (143, Senior).” Shedding light on perceptions of life satisfaction, individual and organisational job satisfaction, self-efficacy, and the types of self-care contributing to these variables is important from an individual perspective, as is how teams and leadership support the wellbeing of people in various roles. A holistic approach to employee wellbeing not only requires a multi-level and interdepartmental approach but also the willingness and commitment to care, listen, and engage to respond to needs and challenges (102). Evidence from other care professions indicates that connection and understanding can, in turn, result in the provision of adequate and meaningful support, increased perception of organisational job satisfaction and overall wellbeing (103, 104).

For the highest *Education*, BSc was the most reported (*n* = 236, 53%), which aligns with the findings of Bunderson and Thompson (2). However, while a university degree in biology, psychology, or zoology is a good foundation and a more common prerequisite to obtain a ZOAQ job, it is important to acknowledge these degrees do not align with the knowledge, skills, and tasks required in various ZOAQ job roles. Therefore, transitioning into a professional animal caregiver profession and up through the ranks will require a considerable amount of additional training, time, effort, and resources as these degrees do not closely align with professions and activities conducted by animal caregivers, curators, or other zoo positions (105, 106). This is likely one of the reasons that it takes people in the field a long time to feel capable and comfortable in their role and explains some of the growing pains alluded to in the interviews. This also likely explains why *Experience* plays a crucial role in all the three variables, and why *Education* was not retained as a predictor (it was not significant) for organisational job satisfaction and self-efficacy. This is an important finding, underscoring that the zoo and aquarium field requires further professionalisation, standardisation, and regularisation to align job roles with professional and meaningful degrees which prepare people for this field. While still largely unregulated and unstandardised, the zoo field have seen an ongoing shift in the types of requirements to obtain a zoo job, such as animal caregiving (51). Veterinarians and veterinary professionals are the only zoo professions in which a degree and certification are mandatory to fulfil the job requirements, and whose educational pathway aligns more closely with their job roles. A BSc degree is followed by higher education certification/vocational; however, it is important to note that many middle and higher education certification/vocational degrees vary considerably in content and are often still largely agriculturally based.

With respect to the need for further training, recommendations for zoo leadership by Bacon et al. (107) highlight the need for engagement and appropriate resourcing of animal behaviour and welfare training for their staff. They underscore the importance of professional education, which includes comprehensive information on the ecology and behaviour of zoo species, alongside more traditional animal welfare education. Related to our findings, other important skills and capacities, such as ways of communication and collaboration, professional and personal self-care, dealing with grief and loss, and celebrating and growing resilience, are all crucial to effective and protective employee wellbeing, but are mostly lacking (105, 106). Finally, Desmet and Ogle (108) reported that providing access to training and information on animal welfare often resulted a more positive perception of their employer and overall view of zoos meeting the Five Freedoms with captive felids, as well as higher job satisfaction in felid keepers.

Our results of perceiving the job as a calling align with Bunderson and Thompson (2). While they did not begin their investigation with a focus on work as a calling, 91% of the zoo staff interviewed (*n* = 23) reported a sense of calling. Like their study, 58.6% of the participants in our study considered their job to be calling/passion. No relationship was found between calling and age, which may be explained by the fact that people can enter the profession and follow their passion at any stage of their life, depending on opportunity and accessibility, considering how hard it is to enter the field. Some may start early when they have little responsibility and expenses, whereas others may start later in life when they are in a relationship, have more experience, or have extra savings. The predominant age bracket in this study, ranging from to 31–35 years, may be reflective of some of these reasons.

“We have our mission statement and overall, we do a good job directing our efforts. I'm here for the animals, really proud of the work, but sometimes it feels a little bit like taking advantage of the passion. We want to provide the best care for the animals, so sometimes that comes at our cost and maybe we don't feel supported in the right ways” (126, Senior)

Interestingly, while individual and organisational job satisfaction showed a similar relationships with self-efficacy, self-care and life satisfaction, we found a meaningful difference in how they relate to calling. Indeed, calling was positively associated with individual job satisfaction but not with organisational job satisfaction. More research is needed to understand the concept of calling and how it influences both aspects of job satisfaction slightly differently. In particular, more light needs to be shed on what does calling specifically mean, to what do individuals feel called, what do they care about? Finally, we need to be careful how we measure, for example individual job satisfaction because measuring with just the single item, might reveal entirely different results for some variables than when assessed with variables, or may not make a difference, all are important to think about before starting the research.

Only a small majority of the participants across different professions and with diverse experience levels (66.1%, *n* = 292) considered themselves to be in a helping profession. This is somewhat surprising, as people in animal care and other job positions are driven to achieve goals such as education, research, and conservation, which require helping in a variety of ways. Caring for animals, guest and conservation programs, animal caregivers, curators, directors, and others, all interact with each other and must, to a certain extent, care for each other to work towards collective goals. These professionals also need to care for themselves so that they can be well while trying to do good. It is important to understand that caring can bring about opportunities, joy, and negative stressors (1, 51, 106), burnout (109), or grief and loss (110). Perhaps as it is a mostly unstandardised and unregulated field where caring for animals and each other is not systematically taught, the awareness of being in a helping profession or perception of divers from the ‘standard’ helping profession such as being nurse or doctor. Awareness and acknowledgement of being in a caring profession directs attention to the required knowledge, practices, and ongoing support and training needed to be well while doing good (1, 51, 52, 111).

The single question for individual job satisfaction was strongly correlated with organisational job and identified that most participants had high levels of individual job satisfaction (i.e.,44.3%, *n* = 196) of the participants responded that they *somewhat agreed*, followed by *strongly agreed* (27.4%, 121). Considering that the majority was reasonably or positively satisfied with their jobs, it reflects that certain aspects of the work and support are aligned with participants’ values and expectations, and the need to, for example, deliver good animal care and have a good team to work with. However, we acknowledge the limitation of using a single question (even if validated) as individual job satisfaction is a complex subject influenced by a wide array of factors such as opportunities for growth, a sense of purpose, work-life balance, and compensation. We combined various questions in the survey and interview to gain more insights on the aforementioned factors which could not be captured within 1 question. Further conversations and enquiries are needed to understand and improve the conditions of those who reported somewhat agree or even lower. To this end, we explored the individual job satisfaction scores per job position (see Figure 1), which showed that for Juniors, the score distribution covered the entire scale, and a large variation in score distribution was also observed for Seniors and Curators. As most of those who *neither agreed nor disagreed* were Juniors, this group of people could be on the fence of whether to stay in the profession or at the facility, while those who somewhat disagree and strongly disagree could be on their way out. Low job satisfaction has been associated with poor workplace culture, contributions to toxic work environments, high turnover, breakdown of communication, and hindered collaboration (9, 112). These findings may account for the large range of job satisfaction among Juniors, who are also the group with the least amount of *Experience*. In contrast, high job satisfaction is associated with work performance, service quality, patient safety, and productivity (113) which may account for those with more *Experience*, Workplace self-care, Intellectual self-care, and self-efficacy, such as the CEOs.

They've got to be able to make a living by showing up here, a pay check for a place to live, food and transport themselves … So, salary and benefits is obviously a core, but I really think regardless of how well they're paid, if they're not valued, and that means listening any participant in a group activity, whether they're monkey keepers, registrars or others…if they're just doing what they're told, I don't think there's much job satisfaction (400, Veterinarian)

Concerning the question related to *work-life and personal life balance*, participants across all job positions reported *work-life and personal-life balance* to be *balanced more towards work* (see Supplementary Table 2). Considering the amount of work in ZOAQs (i.e., balancing caring for animals, fundraising, or animal welfare data analysis), it is not surprising that most participants indicated that integration leans more towards work. Moreover, previous work showed that the nature of working with living beings for those in direct caring professions such as caregivers and veterinary professionals will result in people thinking of their work when going home, wanting to finish this ‘last thing’, or do something extra to improve animal wellbeing (1, 51). Considering that all veterinarians and veterinary professionals did not report *WL and PL integration to be balanced towards life,* this may be due to the medical nature and potentially more acute emergency needs in their daily work. Veterinary professionals are usually also fewer in number, working in smaller teams which may add additional strain and workload, as reported in specialist animal hospitals (114, 115).

Concerning the question on work-life and personal-life integration, participants across all job positions reported integration to be largely satisfactory (see Table 1). However, a large group of people, particularly Juniors and Seniors, indicated that their integration was problematic. Understaffing and overwork have been reported among animal care professionals in ZOAQs, resulting in burnout and other mental health concerns (1, 109, 116), which may account for the current findings. Other explanations may be related to encroachment on family and friends’ time, activities, and duties as reported in other professions such as challenges with childcare (117), impact on relationship quality (118), negative work-home interference (119), and reduced self-care time and opportunities (120). Perceived organisational support and WL-PL balance have a positive direct effect on adaptive performance (121). Self-caring leaders were found to report more staff care than those low on self-care, with their employees perceiving higher staff care while reporting lower strain and better health (122). All contribute to WL-PL integration, and establishing, maintaining, and growing important capacities in a dynamic, potentially stressful, and complex work environment, such as ZOAQs.

## 6 Strengths and limitations

To our knowledge this is the first study to explore the relationships between life satisfaction, individual and organisational job satisfaction, and self-efficacy, and how self-care factors contribute to these variables. Strengths include a good sample size, covering a range of professional roles, and the mixed methods design, which provided a nuanced interpretation of the quantitative findings. There may be some potential bias by the selection process with only 23 large and accredited ZOAQs in English speaking countries. There are no data on the distribution of roles in large ZOAQs, so it is not clear whether our sample is representative of the distribution of staff roles within ZOAQs, but there was a sufficient sample size in each of our categories to generate meaningful results. This study used only age-brackets. ZOAQ employee wellbeing and the wellbeing of animals in their care may also be linked, but this was not explored in this part of the study.

## 7 Conclusion

This research sheds light on how professionals with different roles in ZOAQs - who perceive their work as meaningful and a calling - perceive their life satisfaction, individual and organisational job satisfaction, and self-efficacy, and which types of self-care contribute to these variables. Many participants in this study reported high levels of individual and organisational job satisfaction, but large variations were found between job positions. Many professionals closely responsible for animal care and wellbeing would benefit from further improvements in individual job satisfaction. Individual job satisfaction and calling were correlated, but no relationship was found between calling and organisational job satisfaction, which may suggest a misalignment of values, expectations, and needs. Additionally, based on the results of *Experience* being retained as a predictor for life satisfaction, organisational job satisfaction, and self-efficacy, while *Educatio*n being non-significant, we recommend that the ZOAQ field needs to further develop and influence the type of appropriate, meaningful, and aligned educational pathways, including continued professional and personal development, needed for those in different job positions. This would include the most up to date access to topics such as animal care and wellbeing, caring for self, communication, cross departmental teamwork, and enhancing self-efficacy. Alignment with educational organisations can further professionalise and standardise the field while concurrently working with governmental organisations to regulate the field and professions. These would all contribute to preventing and proactively addressing the needs and requirements do be a professional in ZOAQs, preparing, and continuing supporting individuals for the field and job(s) ahead, which is dynamic in nature.

Foundational education combined with continued professional and personal development can all assist in contributing to a more well-rounded approach to wellbeing, and increasing meaningful, relevant, and empowering experience. Furthermore, overall organisational support, promoting holistic employee wellbeing approaches where people are respected and heard, and seen as bringing value to a mission-driven organisation, compassionate leadership, and a true culture of care will all be essential to support professionals to be well while doing good. Yangming (123) expressed in his work on Unity of Knowing and Acting a sentiment along the lines of ‘To know and not to act, is not to know’. We hope that the knowledge gained will provide stepping stones to act and change. Both individual and organisational commitment and responsibility to self-care and We-care is needed to support life satisfaction, individual and organisational job satisfaction, and self-efficacy and achieving our interconnected collective goals (124) of caring for peoples, other animals, the community of life, and planet we share.

## Data Availability

The data, edited to protect the identity of the participants, supporting the conclusions of this article will be made available by the authors, without undue reservation after PhD completion and lifting of the related embargo.
