# Supplementary material for: The role of self-care in perceptions of satisfaction with life, organisational job satisfaction, and self-efficacy in zoo and aquarium professionals
Source: Front Vet Sci. 2025 Dec 5;12:1677195. doi: 10.3389/fvets.2025.1677195 (PMC12716087; doi:10.3389/fvets.2025.1677195)
Supplement: Supplementary file 1 [file Table_1.docx]

**SURVEY**

| **Question number** | **Question** | **Scale** | **Reference/source/type** |
| --- | --- | --- | --- |
| Q2 | **My job position is (**Please select 1) | Junior animal caregiver  Senior animal caregiver  Curator  Veterinarian  Veterinary professional e.g., veterinary nurse  Animal welfare scientist/coordinator  CEO  Other e.g., nutritionists  Prefer not to say  Optional: Comments | Demographics  (Brando et al., 2023) |
| Q3 | **Please identify your gender (Please select 1)** | Female  Male  Nonbinary  Prefer not to disclose  Optional: Comments | Demographics  (Spiel et al., 2019) |
| Q4 | **Please indicate your age using the age ranges (Please select 1)** | 21-25  26-30  31-35  36-40  41-45  46-50  51-55  56-60  61-65  66+  Prefer not to say  Optional: Comments | Demographics |
| Q5 | **I have worked in the animal care and welfare domain for (using time in the field ranges) (Please select 1)** | Less than a year  1-5 years  6-10 years  11-15 years  16-20 years  21-25 years  More than 25 years  Prefer not to say  Optional: Comments | Demographics  (Brando et al., 2023) |
| Q6 | **Please select your highest education level (Please select 1)** | On the job trained  High school  Higher education certification /vocational  BSc  MSc  PhD  Other  Prefer not to say  Optional: Comments | Demographics |
| Q13 | **I consider my current job to be (Please select 1)** | A way to make money only; I would be just as happy doing other work  A job that I have some interest in doing  A career in which I look to remain in the field Primarily a calling; it's my passion  Prefer not to say  Optional: Comments | (Boivin & Markert, 2016) |
| Q18 | **Organisational job satisfaction**  The organisation appreciates any extra effort from me (reworded from The organization fails to appreciate extra effort)  The organisation listens to complaints from me (reworded from The organization would ignore my complaint)  The organization really cares about my wellbeing  The organization cares about my general satisfaction at work  The organization takes pride in my accomplishment at work, remained as originally reported. | Likert scale  Strongly disagree  Disagree  Somewhat disagree  Neither agree nor disagree  Somewhat agree  Agree  Strongly agree  Prefer not to say  Optional: Comments | (Boivin & Markert, 2016) |
| Q23 | **I am satisfied with my job – Single question** | Likert scale  Strongly disagree  Disagree  Somewhat disagree  Neither agree nor disagree  Somewhat agree  Agree  Strongly agree  Prefer not to say  Optional: Comments | (Dolbier et al., 2005) |
| Q24 | **Do you see yourself as being in a helping profession? For the purposes of this survey a helping profession can be is defined as "a profession that nurtures the growth of, or addresses the problems of, an individual’s physical, psychological, cognitive, or emotional well-being." This applies to humans and other animals. (Please select 1)** | Yes  No  Maybe  Never thought about this  Prefer not to say  Optional: Comments | New, inspired by Green Cross Academy of Traumatology (2025) |
| Q35 | **Physical self-care**  **In the last month I...**  Ate regularly (e.g., breakfast, lunch, and dinner) (modified from: Eat regularly (e.g., breakfast, lunch, and dinner)  Ate foods that promote energy and well-being (e.g.., fruits & vegetables) (modified from: Eat foods that promote energy and well-being)  Exercised consistently (3-4 times a week) (modified from: Exercise consistently)  Got acute medical checkups done when needed (modified from: Get medical care when needed)  Took time off when I got sick (modified from: Take time off when sick)  Did hobbies that are fun and enjoyable (e.g., dance, crafts, walk, sing) (modified from: Dance, swim, walk, run, play sports, sing, or do some other physical activity that is fun  and enjoyable  Got 7-9 hours of sleep per night (modified from: Get enough sleep)  Took all work-entitled holidays (modified from: Take day trips or mini-vacations)  New questions:  Digitally unplugged 1 hour before sleep  Made sure I moved regularly so I did not have long stationary periods  Moved questions:  Wear clothes you like (moved to intellectual self-care)  Omitted questions:  Get regular medical care for prevention  Get massages  Take time to be sexual with myself, with a partner | Likert scale  Never thought about that (modified from Never occurred to me)  Never  Rarely  Occasionally  Often  Always  Prefer not to say  Optional: Comments | (Saakvitne & Pearlman, 1996) |
| Q36 | **Intellectual self-care (renamed from Psychological self-care)**  **In the last month I...**  Read outside of work requirements (modified from: Read literature that is unrelated to work)  Tried new things (modified from: Engage my intelligence in a new area, e.9., go to an art show, sports event, theatre)  Wore clothes I like  Improved my ability to say "no" when I want to (modified from: Say no to extra responsibilities sometimes)  Did something in which I am not an expert (modified from: Do something at which I am not expert or in charge)  Said 'no' to people crossing my boundaries (e.g., not working another extra shift)  Moved questions:  Wrote in a journal (moved to emotional self-care)  Make time away from telephones, email, and the Internet (moved to emotional self-care)  Have my own personal psychotherapy (moved to emotional self-care)  Omitted questions:  Be curious  Attend to minimizing stress in my life  Notice my inner experience - listen to my thoughts, beliefs, attitudes, feelings | Likert scale  Never thought about that (modified from Never occurred to me)  Never  Rarely  Occasionally  Often  Always  Prefer not to say  Optional: Comments | (Saakvitne & Pearlman, 1996) |
| Q37 | **Emotional self-care**  **In the last month I...**  Allowed for quality time with others who have a positive impact on my life (modified from: Spend time with others whose company I enjoy)  Re-read favourite books or re-watched favourite movies  Made time for self-reflection (e.g., listen to and recognise thoughts, judgments, beliefs, attitudes, and feelings)  Found ways to demonstrate love for myself (modified from: Love myself)  Unplugged a day per week from digital devices (modified from: Make time away from telephones, email, and the Internet  Expressed my feelings (laugh, cry, etc.) (Combined: Allow myself to cry & Find things that make me laugh)  Wrote in a journal  Engaged in personal therapy when needed (modified from: Have my own personal psychotherapy)  New questions:  Gave self-affirmations and praise or engage in positive self-talk  Allowed myself to accept time, help, advice, etc. from others  Reached out to friends and family in times of distress (inspired from Relationship self-care: Allow others to do things for me)  Communicated needs and wants (when and where needed with whomever) (inspired from Relationship self-care: Ask for help when I need it)  Omitted questions:  Stay in contact with important people in my life  Express my outrage in social action, letters, donations, marches. Protests  Identify comforting activities, objects, people, places and seek them out | Likert scale  Never thought about that (modified from Never occurred to me)  Never  Rarely  Occasionally  Often  Always  Prefer not to say  Optional: Comments | (Saakvitne & Pearlman, 1996) |
| Q38 | **Spiritual/connecting to meaning self-care**  **In the last month I...**  Spent time in nature  Have been open to inspiration  Cherished my own optimism and hope  Was aware of nonmaterial aspects of life  Meditated  Prayed  participated in a community that shares my core values  Contributed to causes in which I believe  New questions:  Made life decisions based on my core values  Engaged with art that lights me up  Omitted questions:  Sing  Have experiences of awe  Identify what is meaningful to me and notice its place in my life  Try at times not to be in charge or the expert  Be open to not knowing  Find a spiritual connection or community  Make time for reflection  Read inspirational literature or listen to inspirational talks, music | Likert scale  Never thought about that (modified from Never occurred to me)  Never  Rarely  Occasionally  Often  Always  Prefer not to say  Comments | (Saakvitne & Pearlman, 1996) |
| Q39 | **Workplace professional self-care**  Allowed for breaks during the day (modified from: Take a break during the workday (e.g., lunch))  Took time to connect with co-workers (modified from: Take time to chat with co-workers  Took quiet space to complete tasks  Set limits/boundaries as needed (modified from: Set limits with clients and colleagues)  Made sure work space was comfortable  Participated in projects or tasks that were exciting and rewarding  Worked with manager and co-workers to balance workload as required (modified from: Balance my caseload so that no one day or part of a day is "too much")  Negotiated needs (benefits, bonuses, raise, etc.) as required  Omitted questions:  Get regular supervision or consultation  Have a peer support group  (If relevant) Develop a non-trauma area of professional interest | Likert scale  Never thought about that (modified from Never occurred to me)  Never  Rarely  Occasionally  Often  Always  Prefer not to say  Optional: Comments | (Saakvitne & Pearlman, 1996) |
| Q40 | **General Self-Efficacy**  I can manage to solve difficult problems if I try hard enough  If someone opposes me, I can find the means and ways to get what I want  It is easy for me to stick to my aims and accomplish my goals  I am confident that I could deal efficiently with unexpected events  Thanks to my talents and skills, I know how to handle unexpected situations  I can solve most problems if I try hard enough  I stay calm when facing difficulties because I can handle them  When I have a problem, I can find several ways to solve it  If I am in trouble, I can think of a solution  I can handle whatever comes my way | I am not at all confident  I am not at all confident  I am somewhat confident  I am quite confident  I am very confident  Prefer not to say  Optional: Comments | (PROMIS® Health Organization, 2008) |
| Q41 | **Satisfaction with Life Scale**  In most ways my life is close to my ideal  The conditions of my life are excellent  I am satisfied with my life  So far I have gotten the important things I want in life  If I could live my life over, I would change almost nothing | Likert scale  Strongly disagree  Disagree  Somewhat disagree  Neither agree nor disagree  Somewhat agree  Agree  Strongly agree  Prefer not to say  Optional: Comments | (Diener et al., 1985) |

**INTERVIEW**

1. Share how you feel supported in your job by your organisation.
2. What are the most reoccurring issues in your organisation that are not being solved for you?
3. Share how you feel supported at work to care for your own wellbeing?
   1. Individual level
   2. Team level
   3. Leadership level
   4. Organisational level

**REFERENCES**

Boivin, G. P., & Markert, R. J. (2016). Factors Affecting the Vocational Calling of Laboratory Animal Care and Research Employees. *Journal of the American Association for Laboratory Animal Science: JAALAS*, *55*(6), 769–774.

Brando, S., Rachinas-Lopes, P., Goulart, V. D. L. R., & Hart, L. A. (2023). Understanding Job Satisfaction and Occupational Stressors of Distinctive Roles in Zoos and Aquariums. *Animals: An Open Access Journal from MDPI*, *13*(12), 2018. https://doi.org/10.3390/ani13122018

Diener, E., Emmons, R. A., Larsen, R. J., & Griffin, S. (1985). The Satisfaction With Life Scale. *Journal of Personality Assessment*, *49*(1), 71–75. https://doi.org/10.1207/s15327752jpa4901_13

Dolbier, C. L., Webster, J. A., McCalister, K. T., Wallon, M. W., & Steinhardt, M. A. (2005). *Reliability and validity of a single-item measure of job satisfaction—PubMed*. https://doi.org/10.4278/0890-1171-19.3.194

Green Cross Academy of Traumatology. (2025). *Standards of Care Guidelines*. https://greencross.org/about-gc/standards-of-care-guidelines/

PROMIS® Health Organization. (2008). *PROMIS Item Bank v.1.0—General Self-Efficacy*.

Saakvitne, K. W., & Pearlman, L. (1996). *Transforming the pain: A workbook on vicarious traumatization* (1st ed.). W. W. Norton & Company.

Spiel, K., Haimson, O. L., & Lottridge, D. (2019). How to do better with gender on surveys: A guide for HCI researchers. *Interactions*, *26*(4), 62–65. https://doi.org/10.1145/3338283
